# Impact of recent life events on the health related quality of life of adolescents and youths: the role of gender and life events typologies in a follow-up study

**DOI:** 10.1186/1477-7525-8-71

**Published:** 2010-07-19

**Authors:** Ester Villalonga-Olives, Sonia Rojas-Farreras, Gemma Vilagut, Jorge A Palacio-Vieira, José Maria Valderas, Michael Herdman, Montserrat Ferrer, Luís Rajmil, Jordi Alonso

**Affiliations:** 1 CIBER en Epidemiología y Salud Pública (CIBERESP), Spain; 2 Health Services Research Unit, IMIM-Institut de Recerca Hospital del Mar, Parc de Salut Mar, Barcelona, Spain; 3 Agency for Health Information, Assessment and Quality, Barcelona, Spain; 4 NIHR School for Primary Care Research, Department of Primary Care, Division of Primary Care and Public Health, University of Oxford, UK; 5 Universitat Autònoma de Barcelona (UAB), Barcelona, Spain; 6 Universitat Pompeu Fabra (UPF), Barcelona, Spain

## Abstract

**Background:**

Most studies on the effect of life events (LEs) have been carried out in convenience samples which cannot be considered representative of the general population. In addition, recent studies have observed that gender differences in the health related quality of life (HRQoL) impact of LEs might be lower than believed. We assessed the relationship between LEs and HRQoL in a representative sample of Spanish adolescents/youths, focusing on gender differences.

**Methods:**

Participants (n = 840) completed the KIDSCREEN-27 to measure HRQoL at baseline and again after 3 years (n = 454). Follow-up assessment included the Coddington Life Events Scales (CLES) to measure LEs experiences in the previous 12 months. Respondents were categorized according to the amount of stress suffered. We calculated both the number of LEs and the Life Change Unit (LCU) score, a summary of the amount of stress inherent to the event and the time elapsed since occurrence. LEs were classified as desirable or undesirable, and family-related or extra-family. Effect sizes were calculated to evaluate changes in HRQoL. To assess the impact of LEs typologies, multiple linear regression models were constructed to evaluate their effect on HRQoL.

**Results:**

Girls reported a mean 5.7 LEs corresponding to 141 LCUs, and boys 5.3 and 129, respectively. The largest impact of LEs on HRQoL was observed in the group of boys that reported to have lived more stress (third tertil of LCUs distribution). The linear association between LEs and HRQoL tended to be stronger among boys than girls, but the difference was not statistically significant. The effect on HRQoL was deemed important when undesirable events had been experienced. To have an important impact on HRQoL, 200 LCUs due to undesirable events were necessary in boys. In girls, slightly higher scores were necessary for a similar impact.

**Conclusions:**

A moderate association was found between recent LEs and HRQoL, mainly among those who experienced several undesirable events that correspond to at least 200 LCUs. No gender differences were found in this association. Results may be useful for identifying adolescents with particular health risks, regardless of gender.

## Background

Adolescence and youth are considered periods of development when individuals construct their own identity[1]. These periods include life events (LEs) and transitions[2], which can be either desirable or undesirable. LEs can be stressors and demand a special readjustment to reorganise daily life and they might influence child's development[3-5]. Frequent exposure to LEs during adolescence and youth has been shown to be associated with worse health related quality of life (HRQoL)[6-9], psychosomatic complaints[10-12], poor physical functioning, higher risk of disabilities, and greater use of health services[13].

Most studies on the effect of LEs have been carried out in convenience samples which cannot be considered representative of the general population[14-16], limiting their ability to make inferences based on the available observations. Studies based on general population are deemed needed[13,17], to establish whether gender-based differences exist in such samples. Importantly, the associations between LEs and health related outcomes have been generally assessed in cross-sectional design studies [14-16,18]. This design does not allow taking into account health status previous to the LEs suffered, resulting in possible biases. For instance, girls usually report low levels of HRQoL. Lacking a previous measure of perceived health may lead to overestimate the effect of LEs experiences on girls.

Gender differences in exposure and reactions to LEs have been widely discussed[14,15,19,20] and seem to have an effect onto mental health and functional outcomes stronger among girls than boys [15,19,21-23]. But more recent studies have failed to observe gender differences or fewer negative associations. The latter results have reported changes in the traditional gender differences of coping with LEs, might be explained by modifications in social resources, and gender role expectations [2,24-26]. This "buffering hypothesis" would predict a significant interaction effect for social support and life events in relation to psychological outcome, which seems to be the case of girls, which perceive higher levels of social support in recent studies[2]. In addition, although it is known that exposure to LEs can have health consequences, we are not sure about which ones (desirable or undesirable events, or other categories as family or extrafamily) are most influencing[3,4,27].

The aim of the present study was to investigate the impact of LEs on HRQoL using data collected with a longitudinal design, in a general population sample. Additionally, we investigated whether there were gender-based differences in the association between LEs and HRQoL [28]; and whether desirable and undesirable events and those which occurred within the family context versus extra-family impacted differently on HRQoL. We had anticipated that no gender differences in the association between LEs and HRQoL would be found, and that undesirable events and those related to family life would be more strongly associated with HRQoL[3,4,27].

## Methods

### Sample and data collection

The Spanish KIDSCREEN baseline sample was recruited between May and November 2003 as part of the European KIDSCREEN fieldwork [29]. The target population for the KIDSCREEN study was children and adolescents aged 8-18. The aim was to recruit a sample that was representative by gender and age in each participating country according to census data. Telephone sampling was performed centrally from Germany, and was carried out using a Computer Assisted Telephone Interview with random-digital-dialling. Households were contacted by telephone and asked to participate by interviewers who had received study-specific training. If the family member contacted agreed to participate, the questionnaire and other study materials were mailed to the requisite address together with a stamped, addressed envelope and informed consent for return of the completed questionnaire. A telephone hotline was used to provide further information about the survey. Two reminders were sent in cases of non-response (after two and five weeks). More details are provided elsewhere [28,29].

Between May and November 2006, follow-up questionnaires were posted by mail to all adolescents and youths and their parents who had previously agreed to participate in the Spanish KIDSCREEN follow-up study (n = 840 of 926 participants at baseline; 91%). Data collection at follow-up took place 3 years after baseline, a period which was considered a sufficient interval to allow for substantive changes in participants' health status. The fieldwork followed the same methodology applied at baseline. Postal reminders were sent four and eight weeks after the first mailing to those who had not returned their completed questionnaires. A third reminder was sent after twenty weeks and any remaining non-respondents were contacted by phone.

### Measures

#### Life events

Stressful LEs were measured using the Coddington Life Events Scales (CLES)[3,4,27], which measure the occurrence of 53 LEs. Respondents indicate for each item describing a specific LE and the number of times the event has occurred in the last 3 months, 4-6 months earlier, 7-9 months earlier, or 10-12 months earlier. The frequency of occurrence is taken into account in the calculation of Life Change Units (LCUs) which also reflect the amount of stress inherent to the event and how long ago it happened. We used the original LCUs, which were obtained from ratings provided by teachers, paediatricians, and child psychiatrists[3,4,27]. A total LCU score can be calculated for each respondent as a weighted sum of all the LCU scores (range of LCUs for one LE: 5-216). We used the Spanish version of CLES that has been found to be valid and psychometrically equivalent to the original [30].

Life events were classified according to two different typologies: desirable (e.g. "Graduating from high school) vs. undesirable events (e.g. "Divorce of parents) and family-related (e.g. "Loss of a job of your father or mother") vs. extra-family events (e.g. "Going on the first date") [3-5,27,31]. Each LE was classified accordingly into one of the two categories in each typology, except nine which were classified in only one LE typology (e.g. "Being hospitalized for illness or injury" was undesirable but was not classified in the 'family' typology because it was neutral with regard to that particular typology).

#### Health Related Quality of Life

HRQoL was measured using the KIDSCREEN-27[32], which was administered at baseline and follow-up to the adolescents and youths (self-reported) and to their parents (proxy-reported) with a recall period of 1 week. The KIDSCREEN-27 has 5 dimensions: Physical Well-being (PH, 5 items); Psychological Well-being (PW, 7 items); Parent Relation & Home Life (PA, 7 items); Social Support & Peers (PE, 4 items) and School Environment (SC, 4 items). We also calculated an overall index score (KIDSCREEN-10) based on selected items[32].

The KIDSCREEN items use 5-point Likert-type scales to assess either frequency (never-seldom-sometimes-often-always) or intensity (not at all-slightly-moderately-very-extremely). Rasch scores are computed for each dimension and for the overall score and are transformed into T-values with a mean of 50 and standard deviation (SD) of 10. The T scores refer to the mean values and SD from a representative sample of the European general population so that scores over (or under) 50 indicate better (or worse) HRQoL than the general EU population. The Spanish version of the questionnaire has demonstrated acceptable validity and reliability [33,34]. In this study, only responses from adolescents and youths on the KIDSCREEN questionnaire were used.

#### Pubertal development

Pubertal development was measured in order to adjust for possible differences between boys and girls in terms of pubertal status[1] and because of its demonstrated relationship with HRQoL[35]. We used the Pubertal Development Scale (PDS), a self-reported measure with acceptable levels of validity [36], which assesses pubertal characteristics. Subjects respond to each item on a 4-point ordinal scale (no development = 1, development barely begun = 2, development definitely underway = 3, and development already completed = 4). An extra response category was included in each item of the PDS to determine whether development had been completed before the baseline assessment. The menarche item was scored 1 if the girl was pre-menarche and 4 if menstrual periods had already begun. Item scores were summed to produce an overall continuous score ranging from 5 to 25. Higher scores reflect a greater degree of pubertal development. Pubertal development was only measured at follow-up.

#### Sociodemographic variables

Other variables collected in the present study to characterize the sample were family socio-economic status and parental level of education. Socio-economic status was measured using the Family Affluence Scale[37], which includes questions on family car ownership, having own unshared room, the number of computers at home, and how many times the family went on holiday in the previous 12 months. FAS scores were categorized as low (1), intermediate (2), and high (3) affluence level. Parental level of education was collected from the adult respondent and included the highest family level of education according to the International Standard Classification of Education (ISCED). Categories were: low (a maximum of lower secondary level, ISCED 0-2), medium (upper secondary level, ISCED 3-4), and high (university degree, ISCED 5-6)[38].

### Statistical analysis

Differences between boys and girls in relation to LEs and HRQoL were tested using independent two-sample t-tests for continuous variables and chi-square tests for categorical variables. P-values were adjusted with the Hochberg method in order to address the multiple testing problem. The decision rule is to reject the null hypothesis when the adjusted p-value is less than α = 0.05[39].

To investigate the first aim of the study, bivariate analyses of the effects of LEs on HRQoL dimensions for boys and girls were performed. We calculated the effect sizes of changes in HRQoL (difference between follow-up and baseline scores divided by baseline standard deviation) in three different groups of increasing LEs impact. The three categories were defined based on tertiles of LCU distribution (i.e. 0-67 LCU (low), 68-160 LCU (medium), and 161 LCU or more (high)). Two way ANOVA was used to determine whether gender differences were statistically significant.

Multiple linear regression[40] models were tested to investigate the second aim of the study of whether LEs typologies impacted differently on HRQoL. The dependent variables were KIDSCREEN dimensions and overall scores at follow-up; independent variables were the LCU scores of global LEs and typologies. A χ^2 ^test to assess non-linearity between LCUs and HRQoL was not significant, and LCUs were therefore introduced as a linear variable in the model. Models were fitted to estimate the relationship between LEs and HRQoL controlling by baseline HRQoL, pubertal development, and age and stratified by gender. P-values were adjusted with the Hochberg method in order to account for the analysis of multiple end-points. We tested gender differences in the association between LEs on HRQoL by running similar models where the gender and the interaction between gender and LCU scores were included and evaluating the significance of the interaction effect with a two-sided significance test at α = 0.05.

Coefficients in the multiple linear regression indicate the units of change in the dependent variable which is associated with 1 LCU suffered by respondents. To give more interpretable results, we selected four examples to illustrate the magnitude of the effect of the coefficients. To do so, the regression coefficients were multiplied by different LCUs values that correspond to selected amounts of LCUs (i.e. 113 LCU, 165 LCU, 235 LCU and 281 LCU) to assess the direct effect on KIDSCREEN. In addition, to determine minimally important differences and moderate important differences between respondents (LCUs necessary to have a change in HRQoL of 0.2 and 0.5 SD, respectively)[41]. This transformation was applied only to undesirable events due to their special impact.

We did not attempt to evaluate age groups differences due to insufficient sample size after distributing participants by LE typology and gender.

### Results and Discussion

At follow-up, 454 families were re-assessed (response rate: 54%). A total of 423 adolescents/youths with complete data were included in the analysis. Mean age was 15.4 (SD 2.84) years and 51.8% were girls (Table 1). When compared with non-respondents at follow-up, respondents were younger with a slightly higher parental level of education. KIDSCREEN scores at follow up were lower (poorer HRQoL) than at baseline for all dimensions. Girls reported significantly lower scores than boys (p < 0.05) in the Physical Well-being dimension both at baseline and at follow-up.

**Table 1:** Sociodemographic and HRQoL characteristics of the study sample.

	Baseline (2003)				Follow-up (2006)		
		Boys N = 204	Girls N = 219		Boys N = 204	Girls N = 219	
		Mean [%] (SD)	Mean [%](SD)	P-value^*^	Mean [%] (SD)	Mean [%] (SD)	P-value
**Sociodemographics**	**Age**	12.1 (2.82)	12.7 (2.86)	0.46	15.1 (2.82)	15.7 (2.86)	0.41
	Pubertal Development**	NA	NA		15.3 (5.33)	18 (4.48)	<0.001
	**Parental level of education**						
	**Low**	[50.5%] (50)	[48.6%] (50)	0.93	[39.1%] (18.8)	[40.0%] (49)	0.98
	**Medium**	[25.5%] (43.6)	[20.7%] (40.6)		[34.4%] (47.5)	[28.3%] (45)	
	**High**	[24%] (42.7)	[30.7%] (46.1)		[26.5%] (44.1)	[31.7%] (46.5)	
	**Socioeconomic level**						
	**Low**	[21.5%] (41.1)	[16.8%] (37.4)		[13.7%] (34.4)	[16.1%] (36.8)	0.98
	**Medium**	[47.5%] (49.9)	[52.3%] (49.9)	0.93	[54.8%] (49.8)	[52.1%] (50)	
	**High**	[31%] (46.2)	[30.8%] (46.2)		[31.5%] (46.4)	[31.8%] (46.6)	
**Health Related Quality of Life (KIDSCREEN)**	**Overall score (KS-10)**	54.5 (10.7)	52.9 (11.9)	0.93	50.5 (8.58)	49.4 (9.42)	0.89
	**Physical Well-being**	54.2 (10.3)	50.4 (11.5)	0.005	50.7 (9.39)	46.6 (10.3)	< 0.001
	**Psychological Well-being**	54.8 (10.3)	52 (11.2)	0.07	51.5 (9.02)	49.9 (9.76)	0.41
	**School Environment**	53 (10.7)	53.5 (10.7)	0.93	50.1 (10.1)	51.8 (8.97)	0.41
	**Parent Relation & Home Life**	53.2 (9.03)	53.1 (10.7)	0.93	51.5 (8.80)	51.5 (9.16)	0.98
	**Social support and Peers**	53.5 (8.90)	53.9 (9.17)	0.93	49.3 (8.27)	52.1 (9.06)	0.06

The Spanish KIDSCREEN follow-up study

* Comparison between boys and girls using t-test for continuous variables or χ^2 ^test for categorical variables. P-values adjusted for multiple testing with the Hochberg method

** Pubertal Development was not available at baseline

Boys reported a mean of 5.3 LEs in the previous 12 months compared to 5.7 for girls (P = 0.98) (Table 2). Desirable events (a mean of 3.1 among boys and of 3.5 among girls) were more common than undesirable events (2.0 and 2.1), respectively. Extra-family events (4.4 in boys and 4.7 in girls) were more common than family events (0.9 in both boys and girls). The mean of Life Change Units was 127.2 (SE 8.15) for boys and 139.2 (SE 8.04) for girls (P = 0.88). Girls tended to have higher LCU scores in all LEs categories, though differences in scores were not statistically significant.

**Table 2:** Life events (previous 12 months) reported by the study sample.

Type of life events	Number of life events (in the last 12 months)			Life Change Units		
	Boys (N = 204)	Girls (N = 219)		Boys (N = 204)	Girls (N = 219)	
	Mean (SE)	Mean (SE)	P value*	Mean (SE)	Mean (SE)	P value*
**Overall**	5.33 (0.35)	5.68 (0.37)	0.98	127.2 (8.15)	139.2 (8.04)	0.88
**Desirable**	3.12 (0.22)	3.46 (0.23)	0.98	65.86 (4.42)	75.92 (4.86)	0.64
**Undesirable**	1.97 (0.20)	2.05 (0.20)	0.98	55.10 (5.02)	59.37 (4.78)	0.92
**Family**	0.91 (0.11)	0.9 (0.09)	0.98	28.46 (3.82)	28.93 (3.20)	0.92
**Extrafamily**	4.37 (0.32)	4.74 (0.35)	0.98	97.95 (6.86)	108.9 (6.84)	0.88

The Spanish KIDSCREEN follow-up study

* Two-sided t-test for independent samples at p = 0.05 significance level. P-values adjusted for multiple testing with the Hochberg method

Table 3 shows KIDSCREEN scores at baseline and follow-up and effect sizes for each of the three LEs categories (tertiles). In general, HRQoL deteriorated over time in all KIDSCREEN dimensions for both boys and girls. However, in the group of boys that reported the fewest LEs the decrease was small, with effect sizes lower than 0.15 for most KIDSCREEN scales. The exceptions were Social support and peers (ES = -0.41) and the Overall score (ES = -0.26). Boys with more than 161 LCUs showed a decline on all KIDSCREEN-27 dimensions (ES from -0.4 to -0.55), except for Parent relation and home life (ES = -0.25). In girls, the pattern was different because the ES observed in each of the KIDSCREEN-27 dimensions were similar across the three LCU groups and under 0.40 in all cases. Gender differences regarding the change in KIDSCREEN scores were not statistically significant, except for the Social Support and Peers dimension.

**Table 3:** KIDSCREEN mean scores and standardized effect sizes between baseline and follow-up, by level of LCUs experienced

LEs experiences in the previous 12 months (tertiles)	KS-10		Physical well-being		Psychological well-being		School Environment		Parent relation and Home Life		Social Support and Peers	
	Boys	Girls	Boys	Girls	Boys	Girls	Boys	Girls	Boys	Girls	Boys	Girls
**Low: 0-67 LCUs (33.3%)**												
**Baseline**	55.3	55.4	52.5	51.7	54.8	54	54.8	55.6	54.1	55.5	53.6	55.2
**Follow-up**	52.3	51.7	51.9	47.9	54	52.1	53.7	52.7	52.7	53.7	49.7	52.2
**Effect Size**	-0.26	-0.37	-0.06	-0.39	-0.07	-0.19	-0.09	-0.28	-0.13	-0.17	-0.41	-0.31
**Medium: 68-160 LCUs (33.3%)**												
**Baseline**	56.5	51.3	56.3	50.6	55.8	51.6	54.1	52.1	53.9	52.2	53.6	53.9
**Follow-up**	51.4	48.9	51.4	46	51.4	48.9	50.1	52.4	52.4	51.1	49.7	51.1
**Effect Size**	-0.48	-0.19	-0.47	-0.38	-0.4	-0.22	-0.36	0.03	-0.18	-0.1	-0.46	-0.37
**High: +161 LCUs (33.3%)**												
**Baseline**	51.5	52	53.9	48.8	53.7	50.4	49.6	52.7	51.3	51.5	53	52.7
**Follow-up**	47.6	47.7	48.6	45.9	48.8	48.7	46.2	50.4	49.2	49.8	48.2	53
**Effect Size**	-0.44	-0.33	-0.52	-0.23	-0.51	-0.16	-0.4	-0.22	-0.25	-0.16	-0.55	0.03
**Overall LCUs (100%)**												
**Baseline**	54.5	52.9	54.25	50.39	54.81	52.02	52.97	53.45	53.16	53.07	53.47	53.93
**Follow-up**	50.51	49.41	50.73	46.62	51.48	49.87	50.12	51.38	51.5	51.52	49.26	52.13
**Effect Size**	-0.37	-0.29	-0.34	-0.33	-0.32	-0.19	-0.27	-0.15	-0.18	-0.14	-0.47	-0.2
ANOVA*	**F (P-value)**		**F (P-value)**		**F (P-value)**		**F (P-value)**		**F (P-value)**		**F (P-value)**	
**Gender (1 df)**	0.17 (0.68)		0.39 (0.53)		1.84 (0.18)		0.94 (0.33)		0.05 (0.82)		7.12 (0.01)	
**LCUs (2df)**	0.35 (0.70)		2.07 (0.13)		1.70 (0.18)		0.44 (0.65)		0.34 (0.71)		0.54 (0.58)	
**Interaction gender & LCUs (2df)**	0.39 (0.68)		1.32 (0.27)		1.28 (0.28)		2.47 (0.09)		0.11 (0.89)		2.09 (0.13)	

*Two way ANOVA

Multiple linear regression analysis indicated that LEs tended to affect more HRQoL dimensions in boys than in girls, though gender differences were not statistically significant (Table 4). After Hochberg adjustment for multiple comparisons, the strongest associations were seen between undesirable events and HRQoL on the Psychological Well-being and School Environment dimensions and the Overall score in boys, and on the School Environment and Physical Well-being dimensions in girls. In the case of desirable, family and extra-family events, none of the coefficients were statistically significant.

**Table 4:** Multivariate analysis of the association of LEs and HRQoL, by life event typologies.

Type of life events	KS-10		Physical well-being		Psychological well-being		School Environment		Parent relation and Home Life		Social Support and Peers	
	Boys	Girls	Boys	Girls	Boys	Girls	Boys	Girls	Boys	Girls	Boys	Girls
**All**	-0.013 (0.005)	-0.006 (0.004)	-0.014 (0.005)	-0.004 (0.005)	-0.015* (0.005)	-0.001 (0.005)	-0.016* (0.005)	-0.007 (0.005)	-0.012 (0.005)	-0.007 (0.005)	-0.004 (0.005)	0.011 (0.005)
***Desirable***	0.000 (0.010)	0.008 (0.008)	-0.013 (0.011)	0.017 (0.008)	0.002 (0.010)	0.005 (0.008)	-0.002 (0.010)	0.011 (0.008)	-0.002 (0.010)	0.007 (0.008)	0.004 (0.010)	0.011 (0.008)
***Undesirable***	-0.024* (0.008)	-0.019 (0.008)	-0.017 (0.009)	-0.025* (0.009)	-0.029* (0.008)	-0.004 (0.008)	-0.031* (0.009)	-0.024* (0.008)	-0.022 (0.009)	-0.02 (0.008)	-0.009 (0.008)	0.013 (0.008)
***Family***	-0.026 (0.010)	-0.016 (0.011)	-0.014 (0.011)	-0.011 (0.012)	-0.029 (0.010)	-0.013 (0.012)	-0.028 (0.010)	-0.011 (0.011)	-0.02 (0.011)	-0.028 (0.011)	-0.02 (0.010)	0.002 (0.012)
***Extrafamily***	-0.007 (0.006)	-0.003 (0.005)	-0.014 (0.007)	-0.002 (0.006)	-0.008 (0.006)	0.002 (0.006)	-0.012 (0.006)	-0.006 (0.005)	-0.009 (0.006)	-0.002 (0.005)	0.003 (0.006)	0.015 (0.006)

Regression coefficients (SE).

* Statistically significant at p < 0.05. P-values adjusted for multiple testing with the Hochberg method.

** KIDSCREEN overall score and dimension scores adjusted for baseline HRQoL, pubertal development, and age.

The impact on HRQoL of an increasing amount of LCUs stemming from undesirable events is presented in Table 5. Data illustrate the magnitude of the effect when several LEs combinations are lived. Two undesirable LEs lived by 16.3% of respondents involve a decrease of 2.72 and 2.14 in KIDSCREEN scores in boys and girls, respectively. While one more undesirable LE is lived, the impact increases considerably. To calculate the minimally important difference (MID) on the KIDSCREEN we calculated the LCUs necessary to have a difference of 0.2 SD, and a moderate important difference established at 0.5 SD. In the case of undesirable LEs the MID of 0.2 is achieved when at least 75 LCUs are lived in boys [weighted life event units 75*(-0.024))/8.58]. A score of 200 LCUs stemming from undesirable events (200*[-0.024]/8.58) would be associated with a decrease of 4.8 points in overall HRQoL in boys which involves a decrement of 0.55 SD in the KIDSCREEN. This value corresponds to a moderate important difference to detect respondents more in risk of health consequences, as reported by previous studies [41] which is the 6.5% of the study sample. In this case, the effects were slightly higher in boys despite differences were not statistically significant, though girls should experience a higher amount of LCUs to have the same effect.

**Table 5:** Estimation of the impact of undesirable events on health related quality of life (KIDSCREEN-10 score)

Total LCUs of undesirable events (previous 12 months)	% of participants	Adjusted estimate (SD) effect on overall HRQoL*			Mean change on KS-10 Overall score		Example of LEs combination
		Boys	Girls	P-value*	Boys	Girls	
113	16.3%	-0.024 (0.008)	-0.019 (0.008)	0.33	-2.72	-2.14	Breaking up with a boyfriend/girlfriend (39 LCUs) Failing a grade in school (74 LCUs)
165	9%	-0.024 (0.008)	-0.019 (0.008)	0.33	-3.96	-3.13	Breaking up with a boyfriend/girlfriend (39 LCUs) Failing a grade in school (74 LCUs) Hospitalization of a parent (52 LCUs)
235	4.3%	-0.024 (0.008)	-0.019 (0.008)	0.33	-5.64	-4.46	Breaking up with a boyfriend/girlfriend (39 LCUs) Failing a grade in school (74 LCUs) Hospitalization of a parent (52 LCUs) Divorce of your parents (70 LCUs)
281	2.9%	-0.024 (0.008)	-0.019 (0.008)	0.33	-6.74	-5.33	Breaking up with a boyfriend/girlfriend (39 LCUs) Failing a grade in school (74 LCUs) Hospitalization of a parent (52 LCUs) Divorce of your parents (70 LCUs) Loss of a job by your father or mother (46 LCUs)

* P-value comparing coefficients among boys and girls

In this representative sample of the general youth population of Spain, we observed a negative association between LEs and HRQoL, especially on Physical Well-being, Psychological Well-being and School Environment, but also on Overall HRQoL. The decrements in HRQoL associated with a higher number of LCUs tended to be greater among boys, though no statistically significant differences were observed between genders. Whereas undesirable events were associated with decrements in HRQoL, desirable events and family and extra-family events were not associated with a corresponding increase or decrease in HRQoL. In particular, the existence of a combination of undesirable events summing to at least 200 LCUs was associated with a sizeable decline in HRQoL (SD 0.5). Our results do confirm the importance of undesirable LEs in HRQoL and suggest that it is not differential by gender, which put forward the importance of a longitudinal design of the study and changes in the traditional gender differences.

These results should be interpreted taking into account several study limitations. First, the response rate at follow-up was 54%. This figure is quite standard for postal surveys [19,42-44] and, importantly, the sample was shown to be representative of the Spanish population in terms of age and gender when compared to census data[28]. A second limitation may arise from the fact that the CLES use an extensive recall period. Although it is conceivable that there may operate a recall bias, we tested the instrument and the recall periods in a pilot study that showed that they were feasible and acceptable to respondents[28]. Thirdly, data on important mediators such as personality and coping styles was not collected and the association described here could be confounded by a number of unmeasured variables. In addition, such confounders could act differently among the different age groups and their inclusion could modify the results. Thus, they should be considered in future studies. Finally, sample size was limited to test age differences. We performed an analysis stratified by age and it showed differences in life events experiences and LCUs scores among males (worse for the older group) but not among females. Also, worse scores in the KIDSCREEN dimensions were observed in older ages, especially in Physical Well-being, School Environment and the KIDSCREEN 10 in females and males. Stratified data analyses are not presented, but are available upon request.

Our study has several strengths. Findings regarding the effect of LEs on HRQoL have almost always been presented from clinical samples [16,45,46], and the present work is one of the few to study the association in a general population sample. Contrary to previous cross-sectional design studies [2,14,47,48], a longitudinal design makes it possible to assess the association between occurrence of LEs and change in HRQoL[14]. In addition, the present work complements a previous manuscript that studies the effect of changes in mental health on HRQoL[49]. There has also been relatively little research into the effects of intra-family and desirable LEs on HRQoL, with most studies focussing on relationships outside of the family, which was previously suggested by T.M. Damush et al[14]. However, we didn't find any difference between intra-family and extra-family events. Despite these results, when the Hochberg method for multiple testing is not applied, as authors like K. Rothman suggest[50], not only undesirable LEs are statistically significant, also family events. Despite of the different recommendations found at the literature, we considered to include Hochberg corrections to have more precise results.

There were HRQoL decreases at follow-up in both gender groups. Girls had lower scores at follow-up[35], but the assessment of the impact of LEs on HRQoL showed no gender differences. These results suggest important differences with previous literature [15,19,21-23]. Previous studies suggested that the burden of demands and limitations on girls was greater due to their role in society which in turn may have made them more vulnerable when adversities are experienced[47]. Previous reports [51,52] found no evidence of differential item functioning (DIF) by gender in the overall KIDSCREEN sample. Thus, gender differences described here must be attributed to real outcome differences rather than biases due to DIF. On the other hand, studies that found less gender differences pointed out that girls may have more sources of support upon than boys than before[25] and experience social reinforcement by turning to friends when they have a problem. Whereas males may experience criticism for not dealing with problems independently[26]. Our observation of higher scores on the social support and peers dimension among girls, although not statistically significant, would support this hypothesis.

The differences we had hypothesized about the effect of the different LEs based on typologies were only partially confirmed. Undesirable events had the most important effect on HRQoL, but contrary to our hypothesis, family events did not affect any of the dimensions, when Hochberg method for multiple testing was applied. Our results are consistent with the literature [53] suggesting the effect of LEs derive primarily from their undesirability. In other words, the negative impact on HRQoL of an undesirable LE with a weight of 50 LCUs will be greater than the corresponding positive effect on HRQoL of having a desirable event with the same weight. However, it is important to note that the association of LEs with HRQoL is moderate and their effect is considerable when several important LEs are lived. In our sample, 16.3% of respondents have a LCU sum score that involves a minimal important difference[41] to consider for clinical interventions (SD 0.2). In comparison with the junior and senior high school of the validation study of the CLE scales [27], the number of LEs reported was similar (5.5 in the previous 12 months). However, in that study, the general population sample had a mean of 177 LCUs[27], which was higher than in our sample (133 LCUs) though that also included the adult respondents.

One important undesirable event is sufficient to be part of the risk group. In the case of the first LEs combination exposed at Table 5 of breaking up with a boyfriend/girlfriend and failing a grade in school involves a sufficient impact to be part of a risk group. In addition, the combination of these two LEs seems to be usual. In fact, not only the LCUs sum score determine the effect, it's the undesirability of these LEs what makes respondents vulnerable after the experience.

Further studies in larger samples would help to confirm or refute our results. Especially, in order to confirm the role of undesirable LEs and the tendency of change of gender differences. Moreover, it should be useful to have different measures of LEs in order to take into account also their effect in previous HRQoL.

## Conclusions

The experience of LEs did impact the HRQoL of adolescents and youths in this sample, although the effect was moderate. Contrary to most previous studies, we didn't find that girls are more in risk than boys in the association between LEs and HRQoL. The occurrence of desirable life events did not produce a corresponding increase or decrease in HRQoL. In our sample, it is necessary to have lived LEs with at least a final sum score of 200 LCUs associated with undesirable events to have a moderate impact. This value may be useful as a cut-point to detect risk profiles in general population which may involve a considerable decrement in perceived health.

## List of abbreviations

LEs: Life events; HRQoL: Health related quality of life; LCU: Life change unit

## Competing interests

The authors declare that they have no competing interests.

## Authors' contributions

JMV, LR, and JA participated in the conception and design of the study. EVO, SRF, GV and JAPV analyzed the data. EVO, JMV, MH, MF, LR and JA participated in the drafting of the article. All authors contributed to a critical revision of the manuscript and made a substantial contribution to its content, and read and approved the final manuscript.
